# The De-Ubiquitinylating Enzyme, USP2, Is Associated with the Circadian Clockwork and Regulates Its Sensitivity to Light

**DOI:** 10.1371/journal.pone.0025382

**Published:** 2011-09-23

**Authors:** Heather Dehlin Scoma, Monica Humby, Geetha Yadav, Qingjiong Zhang, Joseph Fogerty, Joseph C. Besharse

**Affiliations:** Department of Cell Biology, Neurobiology and Anatomy, Medical College of Wisconsin, Milwaukee, Wisconsin, United States of America; Pennsylvania State University, United States of America

## Abstract

We have identified a novel component of the circadian clock that regulates its sensitivity to light at the evening light to dark transition. USP2 (**U**biquitin **S**pecific **P**rotease **2**), which de-ubiquitinylates and stabilizes target proteins, is rhythmically expressed in multiple tissues including the SCN. We have developed a knockout model of USP2 and found that exposure to low irradiance light at ZT12 increases phase delays of USP2^−/−^ mice compared to wildtype. We additionally show that USP2b is in a complex with several clock components and regulates the stability and turnover of BMAL1, which in turn alters the expression of several CLOCK/BMAL1 controlled genes. Rhythmic expression of USP2 in the SCN and other tissues offers a new level of control of the clock machinery through de-ubiqutinylation and suggests a role for USP2 during circadian adaptation to environmental day length changes.

## Introduction

Core clock function in mammals depends on the expression and function of several clock components including CLOCK, BMAL1, the PERs, the CRYs, REV-ERBα, and RORα. During a 24-hour cycle, CLOCK and BMAL1 (ARNTL) heterodimerize and induce the transcription of target genes by binding to E-box elements within their promoter regions [1], [2], [3]. *Pers, Crys, Rev-erbα* and *RORα* are all targets of CLOCK/BMAL1 transactivation. REV-ERBα can directly inhibit *Bmal1* expression while PER and CRY complex formation and nuclear re-entry can inhibit CLOCK/BMAL1 induced transcription [1], [2], [4]. Several additional protein interactions and modifications are required for maintenance of the circadian clock. For example, CK1εδ phosphorylation of PER1 has been shown to precede PER1 degradation via SCFβ^TRCP^ E3 ubiquitin ligase targeting [5]. Hence, protein modifications affecting protein stability are essential for fine-tuning the molecular circadian cycle.

Mammals regularly adapt to changing environments by resetting the endogenous pacemaker in the suprachiasmatic nuclei (SCN) of the hypothalamus. Clock resetting by light involves signaling initiated in the retina and passed via intrinsically photosensitive ganglion cells (ipGCs) to the SCN [6]. Glutamate and PACAP (pituitary adenylate cyclase activation peptide) released from the ipGC terminals induce Ca^2+^ release in the SCN causing multiple changes including phosphorylation of CREB, which binds CRE-elements to induce *Per1* and *Per2* expression [7], [8]. Several clock components can be phosphorylated, sumoylated or ubiquitinylated and degraded through the proteasome pathway [9], [10], [11]. For example, it has been reported that BMAL1 ubiquitinylation follows sumoylation, and is required for BMAL1 transactivation and subsequent degradation [10]. Furthermore, an ubiquitin ligase plays a role during light resetting in *Drosophila* [9], [10], [11], [12], suggesting that ubiquitinylation and de-ubiquitinylation may have a role in responses to light in mammals. Nonetheless, the role of post-translational modifications of clock components in entrainment to light in the SCN is not well understood.

We have identified a circadian de-ubiquitinating enzyme called USP2 (**U**biquitin **S**pecific **P**rotease **2**) with peak mRNA expression at ZT12 in mouse [13] that was later found to be under CLOCK/BMAL1 transcriptional control in SCN [14]. We engineered a targeted mutation of USP2 in mice and found that they exhibit enhanced phase delays at low irradiance levels that have little or no effect on wildtype mice. USP2 forms protein complexes with several clock components, including BMAL1 and can influence the abundance and turnover of BMAL1. Our analysis suggests that USP2 associates with clock protein complexes and stabilizes BMAL1, which in turn regulates sensitivity to early evening light exposure through its effects on transcription of CLOCK/BMAL1 regulated genes.

## Results

### Expression and Oscillation of Usp2 in SCN, Retina and Liver

**Figure 1:**
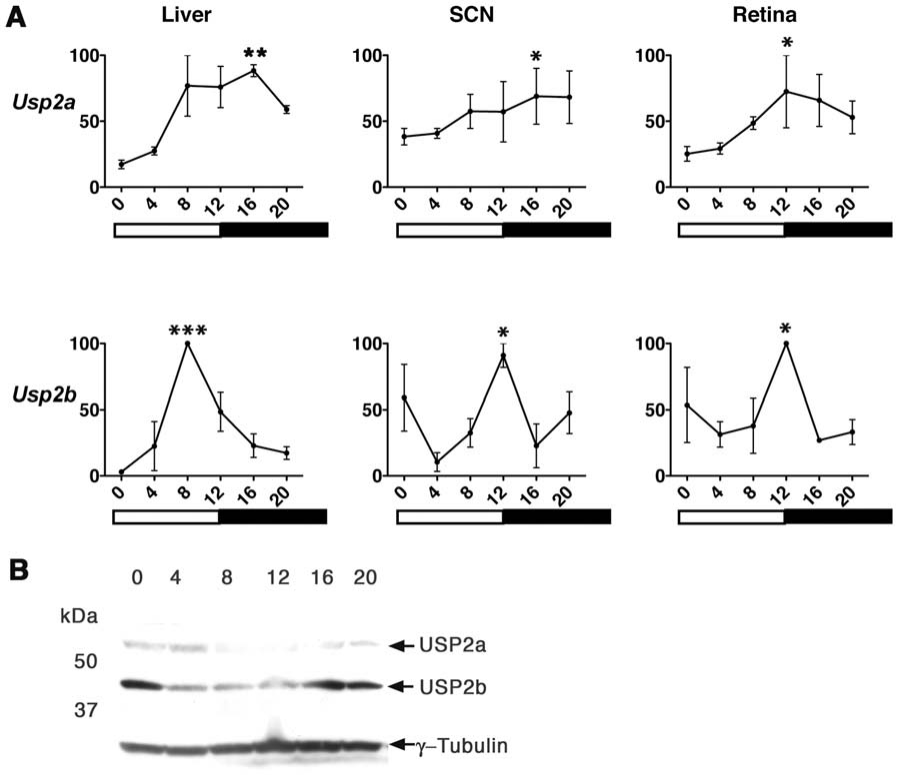
Rhythmic expression of USP2a and b in SCN, retina and liver. **A.** *Usp2a* and ^Usp2b^ mRNA from liver, SCN, and retina of WT C57BL/6 mice kept on a 12-hour LD schedule. Usp2a and Usp2b transcripts were measured by RT-qPCR, expression was normalized to RNA Polymerase II and analyzed using the ΔCT method. The peak was standardized at 100%. The time of day is indicated on the X-axis and with bars. Both transcripts are rhythmic in the liver, SCN, and retina and asterisks signify statistical significance using one-way ANOVA (*p<0.05, **p<0.005, ***p<0.001). **B.** Rhythmic USP2a and USP2b protein levels from SCN of WT C57BL/6 mice housed in LD with γ-tubulin as loading control.

Previous studies [15] have identified two isoforms of *Usp2* that are produced by alternative splicing; a 69-kDa protein (Ubp69 or USP2a) and a 45-kDa protein (Ubp45 or USP2b). Although studies have reported that *Usp2* exhibits a highly robust circadian expression pattern in liver and SCN [13], [14], those studies did not directly compare the two transcripts. Our data show that both forms exhibit rhythmic expression profiles at both the mRNA and protein level. However in its temporal expression, *Usp2b* is the dominant circadian form, exhibiting a peak at ZT8 in liver and ZT12 in SCN and retina (Fig. 1A). Both USP2a and USP2b protein expression changes over time in SCN, but USP2b appears to be more abundant (Fig. 1B). Expression of both USP2a and USP2b is higher at night and early morning (ZT16-ZT0) yet each is detectable in the SCN throughout the day (Fig. 1B).

### Generation and Characterization of the USP2^−/−^ Mice

**Figure 2:**
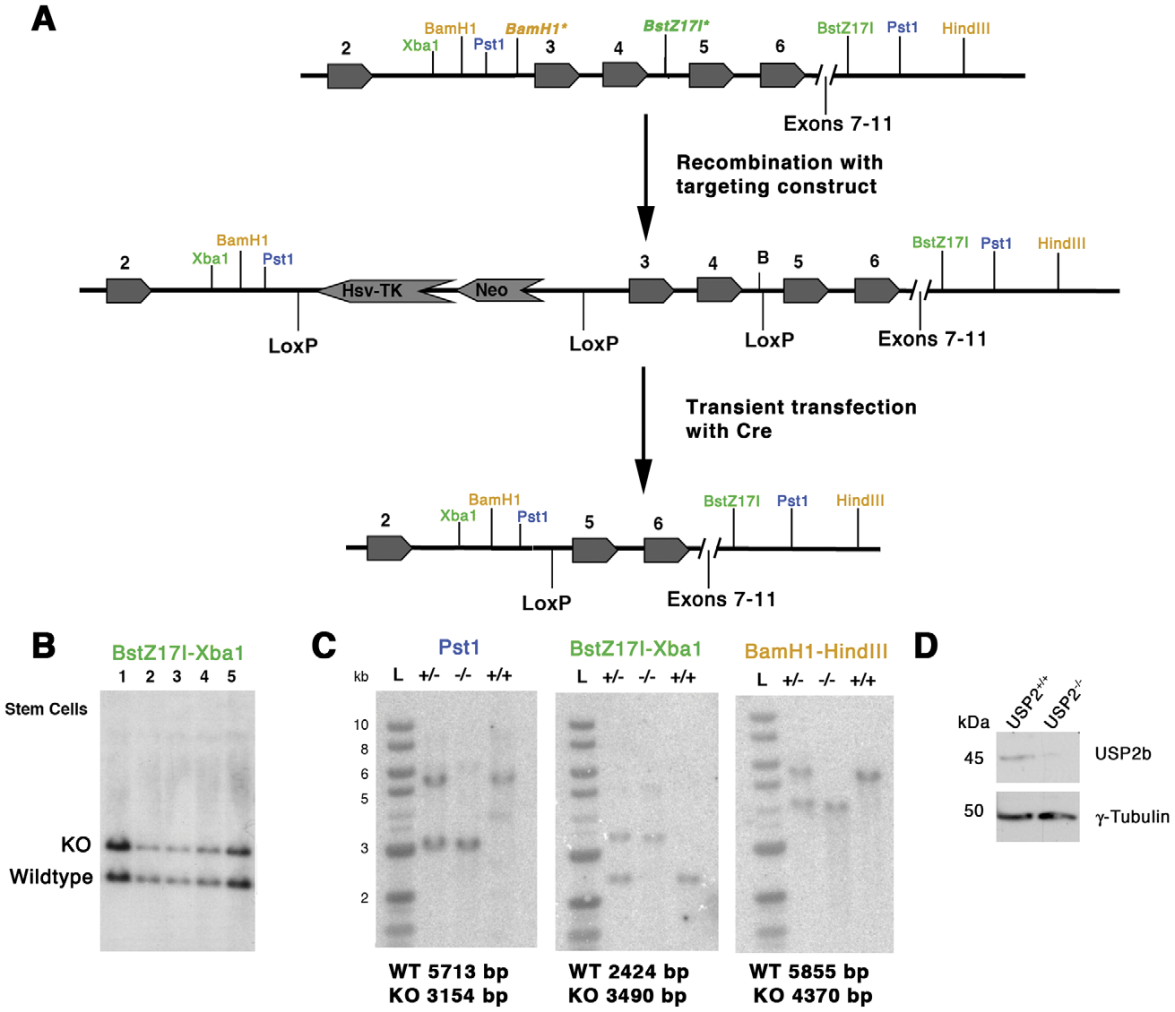
Targeted deletion of Exons 3 and 4 of the *Usp2* gene. **A.** A diagram of a portion of the *Usp2* gene is illustrated at the top with Exon numbering based on Ensembl Gene structure of *Usp2a* (Usp2–201- ENSMUT00000034508). The targeting vector was constructed to introduce LoxP sites upstream and downstream of Exons 3-4 as well upstream of the thymidine kinase (TK) neomycin (NEO) cassette. The structure of *Usp2b* (not shown) begins with an alternative Exon 1 and Exons 3–4 correspond to Exons 2–3 in *Usp2b* (Usp2-202-ENSMUST00000065461). Transient transfection of targeted ES cells with a plasmid encoding Cre recombinase was expected to produce cells carrying a conventional KO allele with a single LoxP site replacing exons 3 and 4 with insertion of a stop codon [39]. Cre expression was also expected to produce a “floxed” allele (not illustrated) with LoxP sites in Intron 2–3 and 4–5 with deletion of the TK-Neo cassette [39]. Three Southern blot strategies for determining correct targeting involved restriction digests with enzymes (color coded) at the relative positions indicated in each diagram. Note that the BamH1 site 5′ of Exon 3 and BstZ17I site 3′ of Exon 4 (each marked by an asterisk) were deleted from the wildtype gene by the targeting strategy. The probe used in the Southern blots in **B** and **C** covered a region spanning spanning Exons 9 through 11 (not shown in the diagrams). **B.** Southern blots of BstZ17I-Xba1 (coded green) digests from five sets of heterozygous stem cells obtained after Cre recombinase transfection and used to generate chimeric mice in the Medical College of Wisconsin transgenic facility. Owing to the loss of a BstZ17I site during targeting the conventional KO allele (3490 bp) is larger than the wildtype allele (2424 bp) using this enzyme pair. **C.** Germ line transmission in mice was obtained only for the conventional KO and is shown by Southern blotting of individual USP2^+/+^, USP2^+/−^ and USP2^−/−^ mice using 3 different restriction digest strategies (Pst1; coded blue, BstZ17I-Xba1; coded green, and BamH1-HindIII; coded orange). Expected sizes of labeled DNA fragments for WT and KO alleles are indicated below each of the three blots. The size of the targeted allele in the BstZ17I-Xba1 and BamH1-HindII digests is caused both by loss of Exons 3 and 4 and loss of a BstZ17I and BamH1 site during targeting. The structure of the targeted allele was verified by genomic DNA sequencing. **D.** Western blot for USP2b and γ-tubulin from the retina USP2^+/+^ and USP2^−/−^ mice at ZT12. USP2a protein was undetectable in USP2^−/−^ mice, but a faint immuno-reactive band is seen in the USP2^-/-^ lane.

We hypothesized that USP2 plays a central role in the circadian clock mechanism. To directly test that idea, we targeted the deletion of exons 3 and 4, which encode a portion of the catalytic region of the enzyme common to both isoforms, and inserted a stop codon (Fig. 2A). The deletion was confirmed in stem cells (Fig. 2B) and in germ line mice (Fig. 2C) by southern blotting. Western blotting from USP2^−/−^ total retina lysate using an antibody generated against the C-terminal region of USP2 showed that USP2a was undetectable. However, we did detect low levels of immunoreactivity at about 45 kDa in USP2^−/−^ retina (Fig. 2D). This could reflect a cross-reactivity or a modified USP2 protein. We used RT-PCR with *Usp2a*- and *Usp2b*-specific primers to determine if any mutant transcripts were produced in retina, liver, and SCN. We failed to detect a transcript using primers for *Usp2a*, but the *Usp2b* primers did reveal a low-abundance transcript. This transcript was cloned and cDNA sequencing not only confirmed the effectiveness of our deletion strategy (Fig.2a) but also showed that Exon 1 of the wildtype Usp2b transcript (Ensembl Gene Identifier ENSMUST00000065461) was alternatively spliced in frame to Exon 5 (see Fig. 2A). These data confirm that a transcript lacking the deleted Exons but encoding the C-terminal antibody-binding site is present at low levels in USP2^−/−^ mice.

### Behavioral Characterization of USP2^−/−^ mice

**Figure 3:**
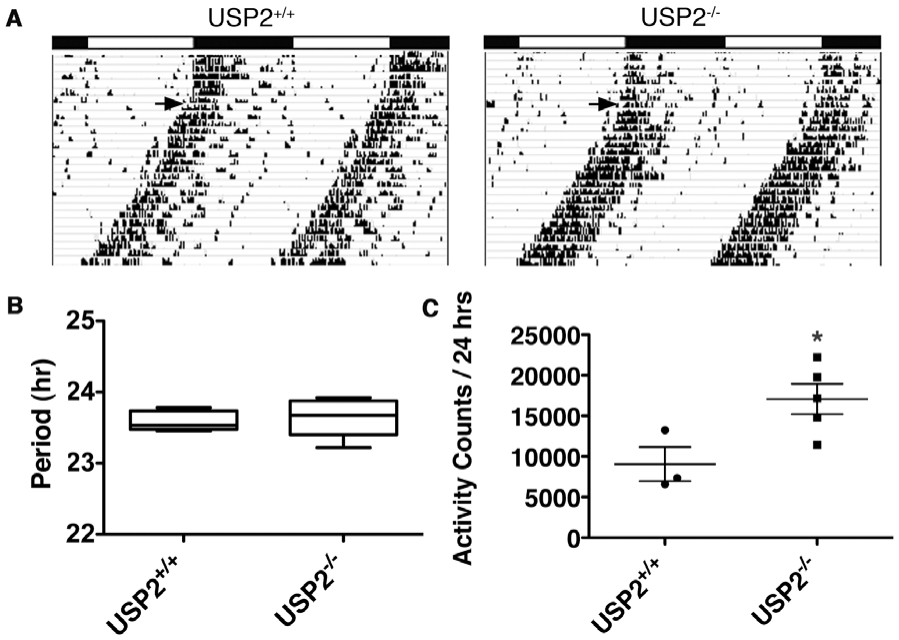
Loss of USP2 does not alter circadian period. **A.** Representative double plotted wheel running records for age matched USP2^+/+^ and USP2^−/−^ mice over 33 days (Y-axis). Mice were initially on a 12-hour LD cycle, followed by a free-run period in DD from day 15 onward (arrow). Data were collected using ClockLab™ software. **B.** The median period measured in USP2^+/+^ mice (n = 5) is 23.53 hours and the 23.68 hours in USP2^−/−^ mice (n = 6). The box represents the percentiles from 25^th^ to 75^th^ and the vertical whiskers represent the maximum and minimum periods measured for each genotype. **C.** Activity counts per 24 hours measured by running wheel revolutions are plotted for USP2^+/+^ (n = 3) and USP2^−/−^ (n = 5) mice housed in constant darkness. The bars are the mean ± SEM and individual data points are plotted for each set; the median activity in USP2^+/+^ mice was 7341 revolutions/24 hours compared to 17,147 in USP2^−/−^ mice. The asterisk signifies a significant difference using the un-paired t-test (p<0.05).

To understand whether USP2 is required for normal circadian function, we used cages equipped with running wheels to determine free-running behavior of USP2^+/+^ and USP2^−/−^ mice in constant darkness (DD) following a period of 12-hour light/dark (LD). Free-running periods measured in USP2^−/−^ mice were indistinguishable from USP2^+/+^ mice (Fig. 3A–B). Although period differences were not found, USP2^−/−^ mice were significantly more active during subjective night when maintained in DD (Fig. 3C).

**Figure 4:**
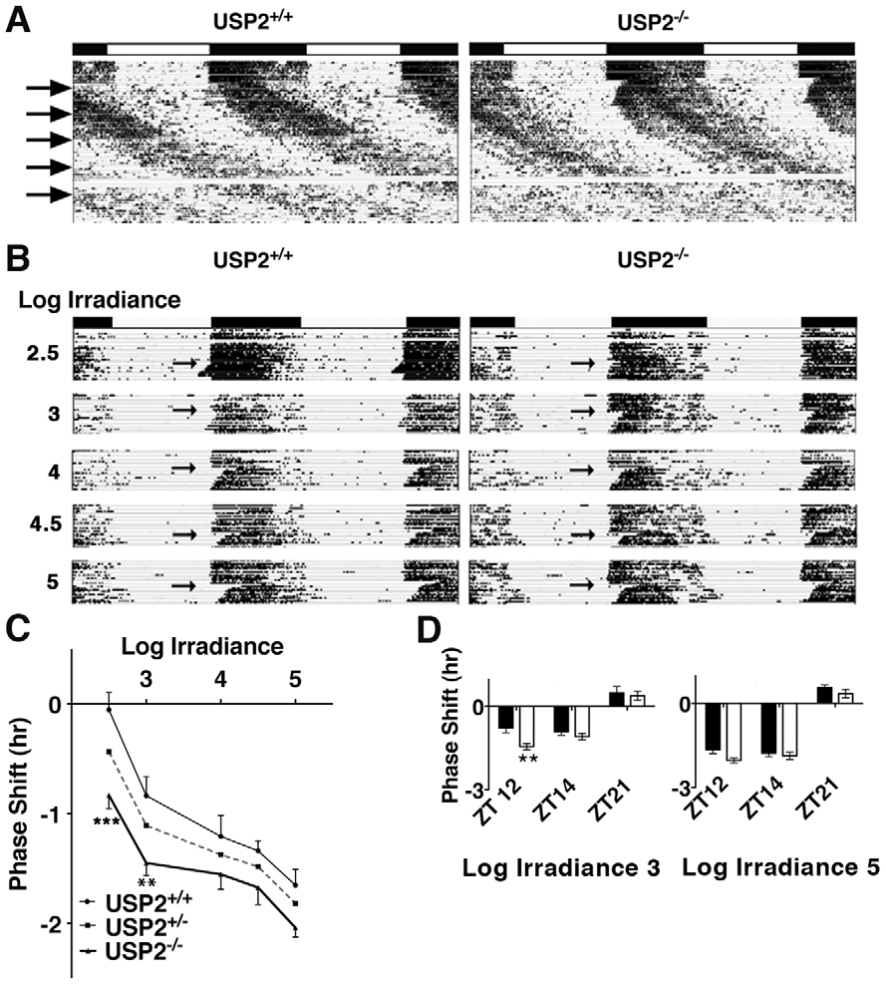
USP2 ^**−/−**^ **mice are more sensitive than wildtype to low irradiance light at ZT12.** **A.** Wheel running records from USP2^+/+^ and USP2^−/−^ mice in constant light. Plots represent the mean activity recorded in USP2^+/+^ (n = 7) and USP2^−/−^ (n = 8) mice. Mice were initially housed in LD (∼60–120 µW/cm^2^) and transitioned to LL (top arrow, left) at ZT12 using low irradiance light (∼5–20 µW/cm^2^). Every 10 days (at each arrow, top to bottom) the irradiance was increased sequentially to 30–60, 50–100, 60–120, and ∼100–160 µW/cm^2^. The white band between the lower 2 arrows is a period when activity was not recorded due to a computer error. **B.** Mice were housed in LD for 7 days followed by a 4-hour dim light treatment (Log irradiance 2.5, 0.05 µW/cm^2^) beginning at the time of light offset and followed by a 7 day free-running period in DD to monitor the resulting phase shift. Mice were returned to LD for 7 days and the experiment was repeated with light of higher irradiance (i.e., Log irradiance 3, 4, 4.5 and 5); Log 5 corresponded to an irradiance of 9 µW/cm^2^. The plots are the mean activity of 5 mice for each genotype. **C.** Irradiance response relationship for USP2^+/+^ (thin line), USP2^−/−^ (thick line), and USP2^+/−^ mice (dashed-line). Phase shifts following each light treatment was compared between USP2^+/+^ and USP2^−/−^ mice (**p<0.005, ***p<0.001, un-paired t-test). **D.** Phase shifts measured at Log irradiance 3 and Log irradiance 5 following 4 hours of light at ZT12 (same data as in C) compared to treatments beginning at ZT14 and ZT21 for USP2^+/+^ (black bars) and USP2^−/−^ (white bars) mice. Significant differences (p>0.05) between genotype were not detected at ZT14 or ZT21.

Ubiquitinylation by the ubiquitin ligase JETLAG in *Drosophila* plays a role during light resetting [12]. We therefore asked whether mice deficient in de-ubiquitinylation by USP2 might also exhibit altered circadian sensitivity to light. First, USP2^+/+^ and USP2^−/−^ mice housed in 12-hour LD were placed in constant light (LL) of low irradiance (∼5–20 µW⋅cm^−2^) and every 10 days the irradiance was increased (Fig. 4A, arrows). As expected, LL increased the period of the activity rhythm and higher irradiances decreased rhythmic activity in both USP2^+/+^ and USP2^−/−^ animals. However, USP2^−/−^ mice were strikingly different than wild type. Their period length increased slowly in response the lower irradiance levels, and, more strikingly, they appeared to exhibit a dramatic delay in activity onset at the lowest irradiance tested. This suggested that the lowest irradiance (∼5–20 µW⋅cm^−2^) was sufficient to cause a phase delay but not sufficient to increase the period length as is expected in LL conditions and as is shown by the control animals.

To determine if loss of USP2 indeed altered the minimum irradiance needed to induce a phase delay at the light-dark transition USP2^+/+^, USP2^+/−^ and USP2^−/−^ mice were entrained to LD conditions and at the beginning of expected light offset (ZT12) exposed to 4-hours of low irradiance light (ZT12–16) followed by 7 days in darkness (DD) (Fig. 4B–C, arrows). They were then re-entrained to the 12-hour LD cycle for a week and the experiment was repeated with light of higher irradiance. USP2^−/−^ mice displayed enhanced sensitivity to light (Fig. 4C) at low irradiance levels (Log irradiances 2.5 and 3 which correspond to ∼0.05 µW⋅cm^−2^ and ∼0.1 µW⋅cm^−2^) and heterozygotes exhibited intermediate phase delays between those of WT and USP2^−/−^ animals (Fig. 4C). Although this trend was apparent across the full range of light levels tested, statistically significant differences were detected only at the lower two irradiance levels in which wild type mice either failed to exhibit any phase shift (Log irradiance 2.5) or exhibited moderate phase shifts (Log irradiance 3). To rule out an influence of genotypic differences in phase angle of entrainment on measured phase shifts this parameter was determined in mice entrained to an LD cycle as above and then released into DD, and differences were not found. Furthermore, the increased sensitivity to light was specific to the paradigm in which light was extended by 4 hours at the time of light off-set (ZT12). Phase shifting was not significantly different in USP2^+/+^ and USP2^−/−^ mice in response to 4 hours of light beginning at either ZT14 or ZT21 (Fig. 4D).

### Altered Clock gene expression in USP2^−/−^ mice

**Figure 5:**
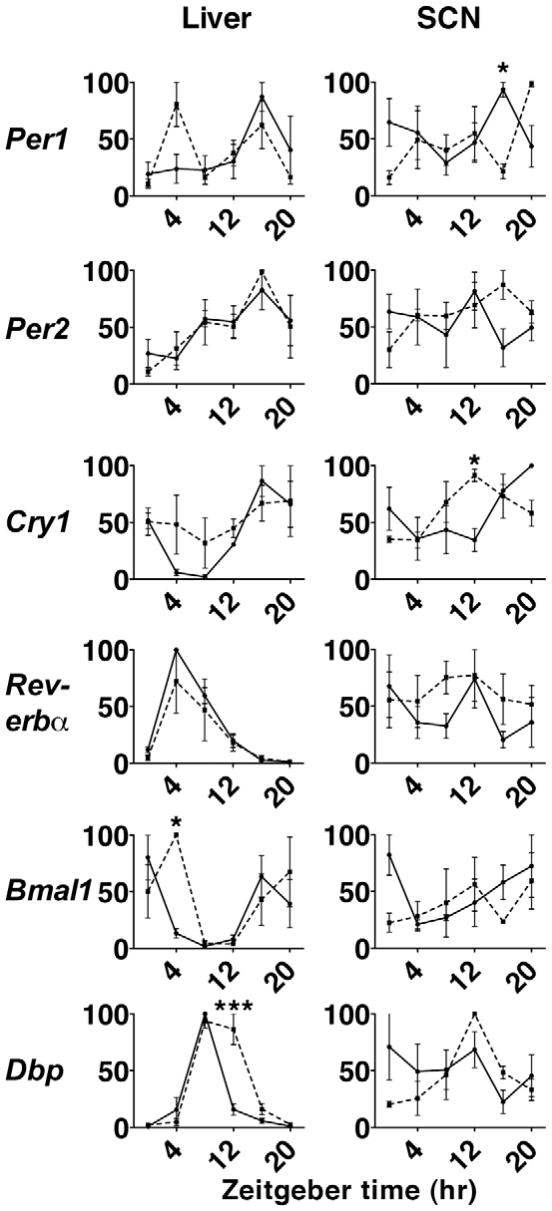
Altered temporal mRNA expression of clock genes in USP2^+/+^ and USP2^−**/**−^ SCN and liver. *Per1, Per2, Cry1, Rev-erbα*, *Bmal1*, and *Dbp* was measured by RT-qPCR and normalized to *RNA polymerase II* expression in SCN and liver isolated every 4 hours from USP2^+/+^ (solid line) and USP2^−/−^ (dashed line) housed in a 12-hour LD cycle. The data was analyzed using the ΔCT method and peak mRNA expression was standardized at 100%. Data points are mean ±SEM (n = 3 animals for each genotype). Two way ANOVA shows significance differences in the rhythms between wildtype and USP2^−/−^ mice (asterisks) for Per1 and Cry1 in SCN as well as BMAL1 and Dbp in liver (*p<0.05, ***p<0.001, two-way ANOVA).

We next asked whether the expression of core elements of the molecular clock were altered in the SCN or liver of USP2^−/−^ mice. The rationale was that such disruption would be expected if USP2 functions within the molecular clock. We began with a quantitative PCR analysis of multiple clock gene mRNAs at different times in an LD cycle (Fig. 5). Using two-way ANOVA for comparison of the patterns seen in USP2^++^ and USP2^−/−^ mice we found that the phase of the peak of *Per1* and *Cry1* expression is 4 hours earlier in USP2^−/−^ SCN. On the other hand, the peak expression of both *Bmal1* and *Dbp* was 4 hours later in USP2^−/−^ liver. Hence, the phasing of expression of some clock components is altered in different ways in USP2^−/−^ SCN and liver. Although these differences suggest that loss of USP2 alters clock activity, it provides no insight into how those changes are mediated.

**Figure 6:**
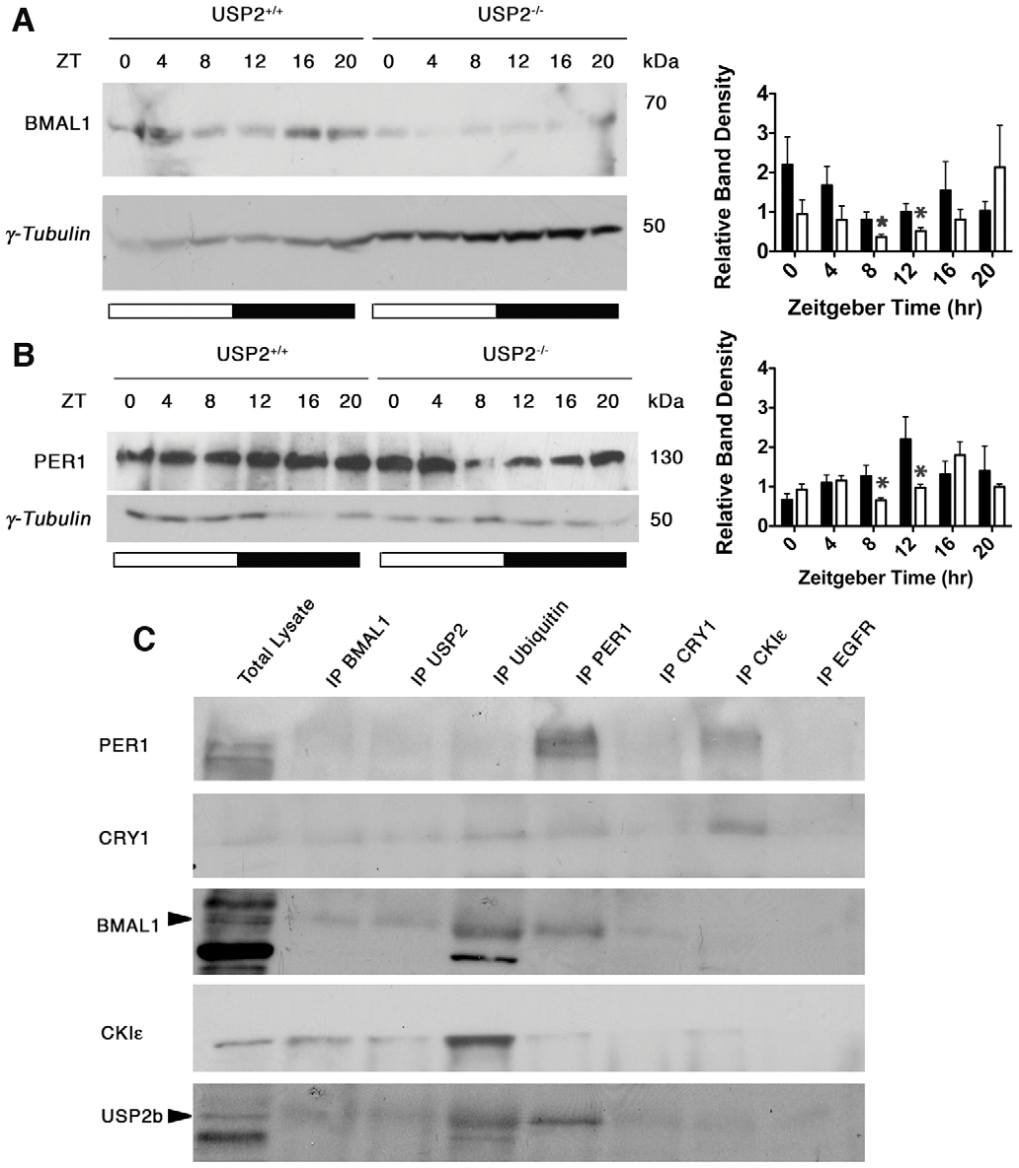
Clock component expression and association with USP2. **A–B**. SCN was micro-dissected every 4 hours from USP2^+/+^ (left) and USP2^−/−^ mice (right) housed in LD. SCN lysate from 5 mice at each time point was pooled (20 µg of protein) and western blotted using either anti-BMAL1 (**A**) or anti-PER1 (**B**) and anti-γ-tubulin antibodies. Band densities were acquired with ImageJ software and plots to the right are mean band density ±SEM for BMAL1 (n = 4) or PER1 (n = 3) normalized to γ-tubulin in either USP2^+/+^ (black bars) or USP2^−/−^ SCN (white bars). Asterisks signify a significant difference (p<0.05) using an un-paired t-test. **C.** Immunoprecipitation (IP) of WT C57BL/6 micro-dissected and pooled SCN extracts (N = 15) at ZT12. IP was performed using protein A beads conjugated with either anti-BMAL1, -USP2, -ubiquitin, -PER1, -CRY1, -CK1ε, or –EGFR rabbit antibodies. IP antibodies are indicated across the top. Western blotting of total lysate (10 µg) and IP samples was performed using rabbit-anti-PER1, rabbit-anti-CRY1, guinea pig-anti-BMAL1, rabbit-anti-CK1ε, rabbit-anti-EGFR, or guinea pig-anti-USP2 antibodies that were different from the IP antibodies. Western antibodies are indicated at left. Arrowheads mark the expected size of either BMAL1 or USP2b.

USP2 encodes de-ubiquitinylating enzymes that stabilize target proteins by removing the ubiquitins of K48-linked chains that flag proteins for proteasomal degradation [16]. Therefore, reduced or altered expression of proteins in USP2^−/−^ mice would provide clues to understanding the molecular targets of USP2 within the clockwork. We began by determining relative PER1 and BMAL1 protein abundance by western blotting in extracts of micro-dissected SCN over the course of a day from mice housed in LD (Fig. 6A and B). First, BMAL1 appeared to be down-regulated in USP2^−/−^ mice at all time points except ZT20. However, the reduction was statistically significant only at ZT8 and 12 (Fig. 6A, right). PER1 was also significantly reduced at ZT8 and ZT12 and its peak abundance was delayed to ZT16 (Fig. 6B, right). These data indicate that both PER1 and BMAL1 are reduced in the SCN at ZT12 when USP2^−/−^ mice are highly sensitive to light-induced phase delays.

Decreased PER1 and BMAL1 expression in the SCN of USP2^−/−^ mice suggests that USP2 might directly interact with protein complexes containing BMAL1 and PER1. To test this we used co-immunoprecipitation to look for protein complexes in wild type SCN protein extracts containing both clock proteins and USP2b, the more abundant USP isoform. We found that anti-BMAL1 and anti-USP2 both co-precipitated small amounts of USP2b along with CRY1, BMAL1 and CK1ε (Fig. 6C). In addition, anti-PER1 precipitated complexes with increased relative amounts of BMAL1 and USP2b (Fig. 6C), while CRY1 and CK1ε antibodies were less effective at bringing down USP2b (Fig. 6C). These data indicate that SCN extracts contain native protein complexes containing USP2b, BMAL1, PER1 and other clock proteins. Interestingly, anti-ubiquitin antibodies co-precipitate CRY1 with larger amounts of BMAL1, CK1ε and USP2b and each protein migrates at its normal molecular weight. This implies that additional ubiquitinylated proteins or ubiquitin chains coexist in complexes containing native, non-ubiquitinylated clock-proteins.

### BMAL1 turnover in the presence and absence of USP2b

**Figure 7:**
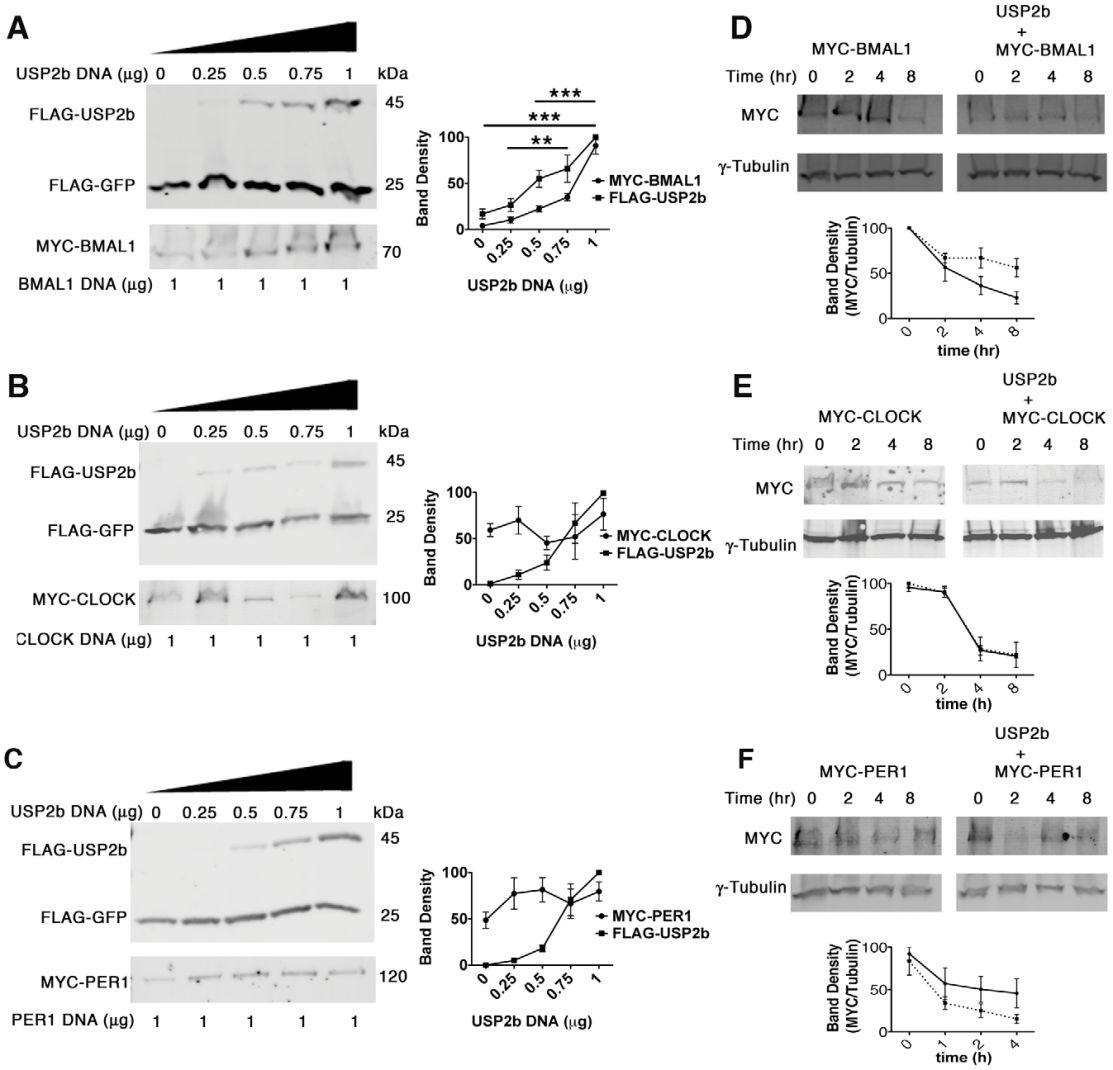
BMAL1, CLOCK and PER1 abundance in the presence of USP2b. **A-C.** Increasing FLAG-USP2b plasmid (0–1 µg) was co-transfected in HEK293 cells with constant (1 µg) bicistronic plasmid encoding FLAG-GFP along with MYC-BMAL1 **(A),** MYC-CLOCK **(B), or** MYC-PER1 **(C)**. FLAG-GFP is constant in each lane but as the concentration of FLAG-USP2b construct increased more MYC-BMAL1 **(A)** was also detected. Plots to the right of each western blot are mean +SEM (N = 3) band density of FLAG-USP2b (squares) or MYC-BMAL1 (circles) (**A**), CLOCK (**B**), or PER1 (**C**). Band densities were normalized to FLAG-GFP as a function of amount of FLAG-USP2b plasmid. One way-ANOVA was used to determine if target protein changed as a function of increasing USP2b. Bars connect points on the plot for BMAL1 that are significantly different from each other and asterisks signify p values (***p<0.0001 and **p<0.001). **D–F.** A bicistronic vector encoding either FLAG-GFP along with MYC-BMAL1 **(D, left)**, CLOCK **(E, left)**, or PER1 **(F, left)**, or FLAG-USP2b along with the same target protein (**D–F**, right) was transfected into HEK293 cells. After 24 hours the cells were incubated with cycloheximide (20 µg/ml) for the indicated periods of time (X-axis). Western blotting of total protein lysates with anti-MYC was used to measure the levels of MYC-BMAL1**,** -CLOCK, and -PER1. Plots below Western blots are mean +SEM for three independent experiments in which target protein band density was normalized to that of γ-tubulin as a function of time in cycloheximide. Solid lines represent co-expression of target protein with FLAG-GFP and dashed lines are with FLAG-USP2b. We used regression analysis to estimate BMAL1 half-life in the absence of USP2b and found that value (3.9 hrs) significantly (p<0.05) increased to greater than 8 hrs in the presence of USP2b. Significant changes were not seen for CLOCK or Per1.

Down-regulation of PER1 and BMAL1 in USP2^−/−^ mice and the presence of USP2b in native clock protein complexes containing PER1 and BMAL1 suggests that USP2b may de-ubiquitinylate and stabilize either PER1 or BMAL1. If USP2b targets PER1 or BMAL1 one would predict that increasing expression of USP2b would increase PER1 or BMAL1 stability and abundance. To test this, increasing concentrations of a FLAG-USP2b construct was transiently transfected into HEK293 cells along with a constant amount of a bicistronic construct expressing FLAG-GFP and MYC-BMAL1. We used one-way ANOVA to ask whether target protein increased significantly with increasing FLAG-USP2b. We found that MYC-BMAL1 rose in parallel with USP2b (Fig.7A), and that the abundance of FLAG-GFP as a control remained nearly constant (Fig. 7A). In contrast, co-expression of increasing amounts FLAG-USP2b with MYC-CLOCK or MYC-PER1 had no significant effect on their protein abundance (Fig. 7B and C). These data indicate that USP2b can stabilize BMAL1, but not PER1 or CLOCK.

To verify these results we used a second approach in which turnover of target, MYC-tagged proteins was determined in the presence or absence of USP2b (Fig. 7D-F). Each MYC-tagged protein was expressed in HEK293 cells with either FLAG-USP2b or FLAG-GFP and after 24 hours protein synthesis was inhibited with cycloheximide and abundance of the MYC-tagged protein was measured over time. Consistent with the effect of increasing FLAG-USP2b (Fig. 7A), the turnover of BMAL1 was delayed in the presence of FLAG-USP2b (Fig. 7D). Using regression analysis we estimated that the half-life of MYC-BMAL1 was about 3.9 hours and significantly (p<0.05) increased to greater than 8 hours in the presence of USP2b. In contrast, USP2b did not significantly (p>0.05) delay turnover of MYC-CLOCK or MYC-PER1 (Fig. 7E and F). Given that BMAL1 is down-regulated in USP2^−/−^ SCN (Fig. 6A) and that its stability is altered by over-expression of USP2b in both assays (Fig. 7A and D) our data suggest that BMAL1 is a likely protein target of USP2b.

### Co-precipitation of USP2b and BMAL1 and de-ubiquitinylation by USP2b

**Figure 8:**
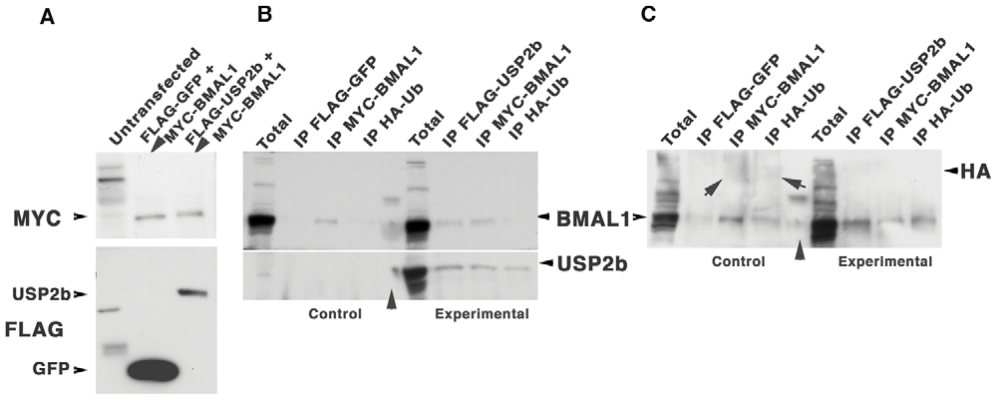
Association of USP2b with BMAL1 and changes in ubiquitinylation. **A.** Expression of control (FLAG-GFP/MYC-BMAL1) and experimental (FLAG-USP2b/MYC-BMAL1) bicistronic vectors in NIH 3T3 cells. Un-transfected lane shows cross-reactivities in the absence of specific antigen. **B.** Reciprocal co-IP of FLAG-USP2b and MYC-BMAL1 and of USP2b with HA-UB in experimental (right) and control (left) transfections. **C.** Upper blot in **B** re-stained with anti-HA-UB to detect ubiquitinated BMAL1 (arrows) in control (left) and its absence in experimental (right) transfection. In **B** and **C** the middle lane (arrowhead) contains MW markers.

To determine if USP2b alters the ubiquitinylation state of proteins in complex with BMAL1, we co-expressed HA-ubiquitin and the bicistronic construct expressing FLAG-USP2b and MYC-BMAL1; as a control we used the FLAG-GFP and MYC-BMAL1 bicistronic construct. We then immunoprecipitated using FLAG or MYC antibodies and analyzed for the presence of BMAL1 (MYC), GFP or USP2b (FLAG), and ubiquitin (HA) (Fig. 8A–C). We first determined that transfection of the FLAG-GFP and MYC-BMAL1 or the FLAG-USP2b and MYC-BMAL1 initially resulted in similar expression levels of MYC-BMAL1 (Fig. 8A). In the control immunoprecipitations (Fig. 8B, left) the FLAG antibody did not pull down MYC-BMAL or HA-Ubiquitin, but the MYC antibody did pull down MYC-BMAL1. In the experimental group (Fig. 8B, right) both FLAG and MYC antibodies co-precipitated MYC-BMAL1 and FLAG-USP2b as expected if USP2b and BMAL1 were present in the same protein complex. In addition, the HA antibody immunoprecipitated FLAG-USP2b, which is consistent with the fact that USP2b can bind to ubiquitin chains. Finally, the MYC-BMAL1 blot in Figure 8B was re-probed with the HA antibody. This generated a smear above the BMAL1 bands in control IPs, but not when BMAL1 was co-expressed with USP2b (Fig. 8C). Thus, the ubiquitinylation of proteins in complex with MYC-BMAL1 was reduced in the presence of USP2b. Taken together with the altered stability and abundance of BMAL1 in the presence of USP2b, this suggests that BMAL1 is a target of USP2b.

### Light resetting in BMAL1^+/−^ and USP2^+/−^/BMAL1^+/−^ mice

**Figure 9:**
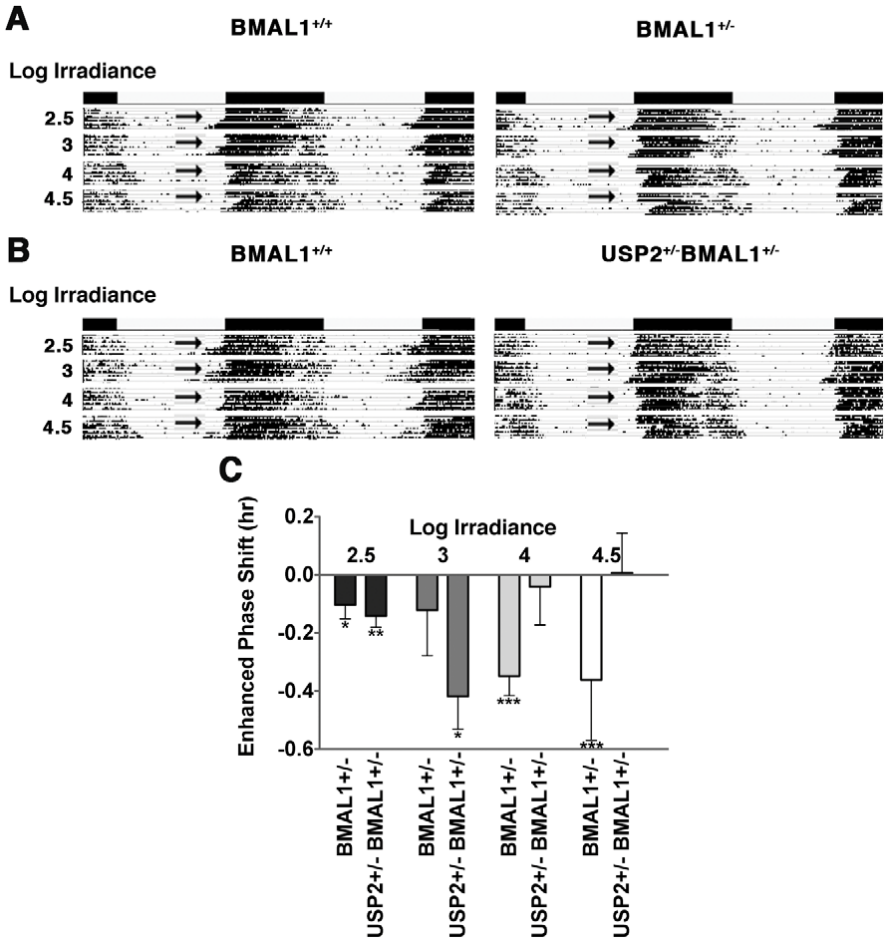
Enhanced sensitivity to light in BMAL^+/−^ and USP2^+/−^/BMAL^+/−^ mice. **A–B.** Mice were housed in LD for 7 days followed by a 4-hour delay in light offset (arrows) and a 7 day free-running period in DD to monitor phase shifts. Mice were then returned to LD and the experiment was repeated with light of a higher irradiance. Actograms shown are averages of 5 individual mice for each genotype. **C.** In this plot phase delays in excess of those in wildtype controls are plotted as the mean "enhanced" phase delay for each mutant genotype (mutant phase delay minus wildtype phase delay) at log irradiance 2.5 (black bars), 3 (grey bars), 4 (light grey bars), and 4.5 (white bars). Means for each genotype exceeded wildtype except for the double heterozygotes at the highest irradiance. Un-paired t-tests were used to compare phase shifts between BMAL1^+/−^ or USP2^+/−^/BMAL1^+/−^ and the wildtype at each irradiance as follows: Log irradiance 2.5; BMAL1^+/−^ and USP2^+/−^BMAL1^+/−^ mice exhibit significant delays in excess of wild type (*p<0.05 and **p<0.005). Log irradiance 3; USP2^+/−^/BMAL1^+/−^ mice phase delayed significantly more than wild type (*p<0.05). Log irradiance 4 and 4.5; BMAL1^+/−^ mice phase delay significantly more than wild type (***p<0.0001).

Since USP2 expression is under circadian control [14] downstream of CLOCK/BMAL1, our work identifies a novel feedback loop at the protein level that would be expected to stabilize BMAL1. However, since USP2 is likely to have multiple targets the relationship between USP2 and BMAL1 does not necessarily explain the enhanced phase delays observed in USP2^−/−^ mice. As a preliminary test of the hypothesis that altered BMAL1 abundance or turnover in USP2^−/−^ mice contributes to the enhanced phase delays at low irradiance, we conducted phase shift experiments like those in Figure 4B using BMAL1^+/−^ and BMAL1^+/−^/USP2^+/−^ mice (Fig. 9). BMAL1 heterozygotes express BMAL1 at about half the level of wildtype mice [17], which is comparable to the reduction seen in SCN of USP2^−/−^ mice at ZT8 and ZT12 (Fig 6A, right). These mice also exhibited significantly enhanced phase delays compared to wildtype in response to light exposure beginning at ZT12 (Fig. 9A–C). However, this was not a precise phenocopy of the USP2^−/−^ effect because the enhanced response was modest at low irradiance (Log irradiance 2.5 and 3) and was more pronounced at higher irradiance (Log irradiance 4 and 4.5).

In contrast, USP2/BMAL1 double heterozygotes were significantly more sensitive to low irradiance light (Log irradiance 2.5 and 3) compared to wildtype, and as in the case of USP2^−/−^ mice, the difference between WT and double heterozygotes was not significant at higher irradiance (Fig. 9C). In response to the two lowest light levels double heterozygotes showed an average phase delay of 38 and 12 minutes less, respectively, than USP2^−/−^ mice and the pattern of phase delays resembled those of USP2^−/−^ mice at low irradiance (compare Fig. 9C and Fig. 4C). The enhanced phase shifts in both BMAL1^+/−^ and BMAL1^+/−^/USP2^+/−^ mice suggest that BMAL1 in USP2^−/−^ mice is at least partially responsible for the enhanced phase delays.

### Molecular clock function in USP2^−/−^ SCN

**Figure 10:**
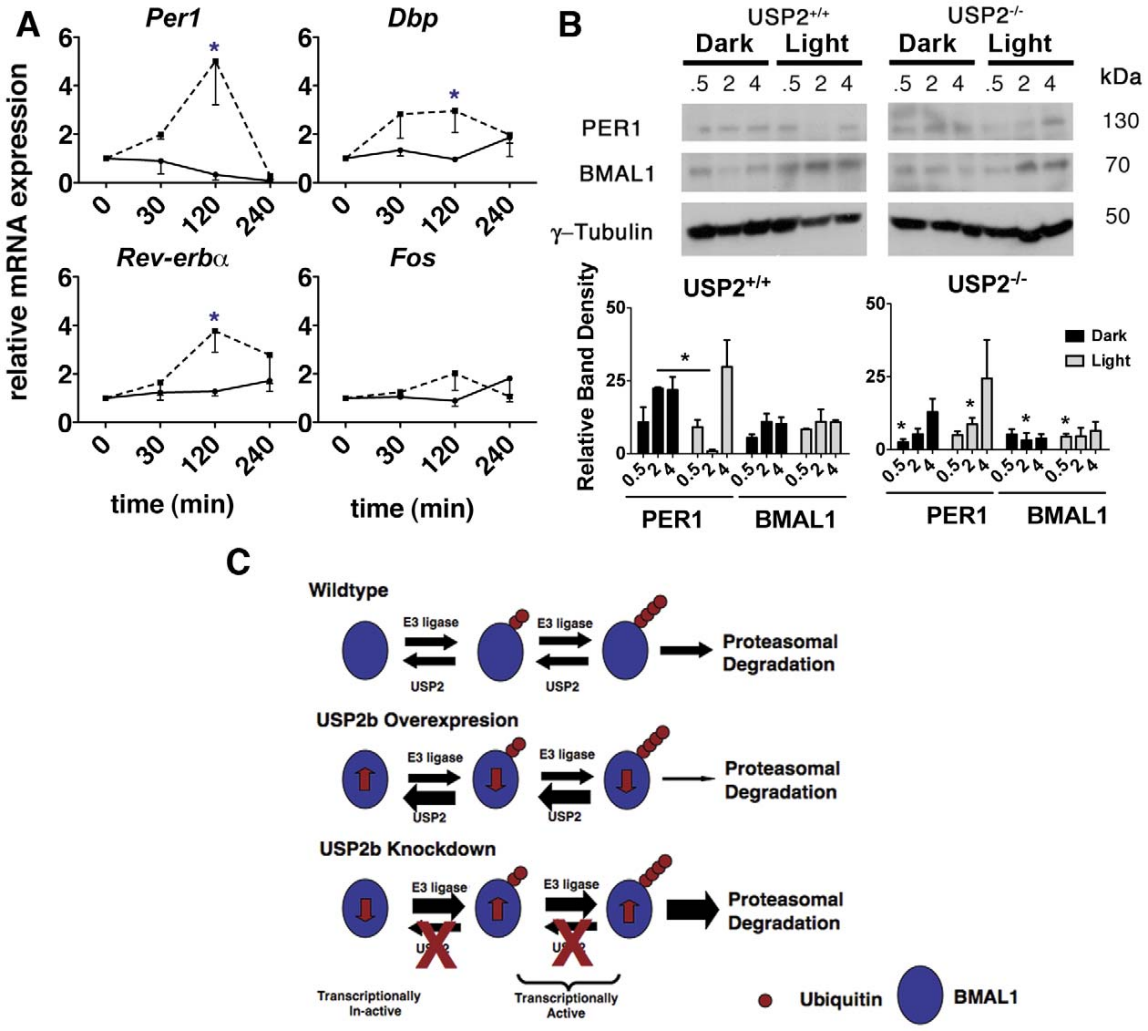
Clock controlled pre-mRNA and protein expression in SCN during low light at ZT12. **A.** Un-spliced mRNA transcripts for *Per1* (Mean ±SEM, n = 3 animals), *Rev-erbα* (n = 6), *Dbp* (n = 6) and *cFos* (n = 3) were measured by RT-qPCR from isolated SCN during exposure to low irradiance light (log irradiance 3) from ZT12 to ZT16. Un-spliced mRNA was normalized to *RNA Polymerase II* and analyzed using the ΔCT method [40]. Changes in each pre-mRNA relative to the zero time point is plotted over time for USP2^−/−^ (dashed line) and USP2^+/+^ (solid line). (Asterisks indicate time points where the difference between genotypes is significant (*p<0.05, two-way ANOVA). **B.** Western blots of PER1, BMAL1 and γ-tubulin in SCN samples at 0.5, 2 and 4 hrs in darkness or low light (log irradiance 3) from ZT12 to ZT16 for USP2^+/+^ (left) and USP2^−/−^ (right) mice. Plots below each set of blots are the mean ±SEM (n = 3) band density of PER1 and BMAL1 (normalized to γ-tubulin) in USP2^+/+^ (left) or USP2^−/−^ SCNs at each of three time points (0.5, 2 and 4 hrs) in darkness (black bars) or low light (grey bars). Note that in USP2^+/+^ mice PER1 normally plummets (to barely detectible levels by 2 hrs of low irradiance light and then rebounds. The horizontal bar connects the 2 hr time point in dark vs. light and the asterisk signifies a significant difference (p<0.05, two-way ANOVA). In contrast, PER1 is on the rise across these time points in light or dark in USP2^−/−^ mice. Significant differences (p<0.05) in protein abundance between genotypes are indicated by asterisks in the plot on right. While BMAL1 abundance does not change significantly with light treatment in either genotype, it is reduced in USP^−/−^ SCNs (see also Figure 6A). Additionally, PER1 is lower at 0.5 hrs in darkness but higher a 2 hrs in light in the USP2^−/−^ SCNs. **C.** Schematic depicting the expected effects of increased or decreased USP2 on target protein (i.e., BMAL1) ubiquitinylation state and abundance. BMAL1 is depicted as being in an equilibrium between enzymes promoting its yet unidentified ubiquitinylation (E3 ligase) and USP2, which promotes its de-ubiquitinylation. Over expression of USP2b would swing the equilibrium to the left stabilizing BMAL1 with less ubiquitin. Loss of USP would swing the equilibrium to the right with reduced BMAL1 abundance and promote higher turnover. Work by others indicates that ubiquitinylated BMAL1 is transcriptionally active but rapidly degraded [10]. Thus, loss of USP2 would reduce BMAL1 levels but also increase its turnover and transcriptional activity. This would include CLOCK/BMAL1 regulated genes such as *Per1*.

How could increased BMAL1 turnover in USP^−/−^ mice contribute to the light sensitivity phenotype that we have described? An important clue comes from the finding that it is the ubiquitinylated form of BMAL1 that is transcriptionally active [10] and that transcriptional activity is closely coupled to BMAL1 turnover [9], [10]. Thus, increased ubiquitinylation and turnover of BMAL1 at the light to dark transition could lead to the increased transcription of CLOCK/BMAL1 regulated genes such as Per1 that are known to be directly involved in delay phase shifts specifically in the lighting paradigm used in our studies. To test this we measured the pre-mRNA expression levels of the CLOCK/BMAL1 controlled genes (*Per1*, *Dbp* and *Rev-erbα*) in USP2^+/+^ and USP2^−/−^ SCN during exposure to early evening low irradiance light (Fig. 10A). We used RT-qPCR with primers designed for un-spliced mRNA rather than processed mRNA because those transcripts are closer to the immediate event of CLOCK/BMAL1 induced transcription. We used *c-Fos* as a control because it is induced by light in the SCN through a pathway that does not involve CLOCK/BMAL1 [18], [19]. *Per1*, *Rev-erbα*, and *Dbp* pre-mRNA were all significantly increased (2–4 fold) in the SCN of USP2^−/−^ mice compared to controls (USP2^+/+^) within 2 hours of low irradiance light (Log irradiance 3) initiated at ZT12, while the pre-mRNA of the immediate early gene, *c-Fos,* was unaffected (Fig. 10A). These data suggest that at the molecular level, the transcriptional activity of CLOCK/BMAL1 in the SCN is enhanced specifically in USP2^−/−^ mice during the low irradiance light treatment.

Next, we asked whether the expression or abundance of BMAL1 or PER1 protein was altered in response to the same light exposure. Western blotting for BMAL1 from protein lysates isolated from USP2^+/+^ and USP2^−/−^ SCN showed no statistically significant effect of low irradiance light but did confirm our earlier data (Figure 6A) that BMAL1 is less abundant in USP^−/−^ mice (Fig. 10B, graph). The striking finding in these experiments was that after 2 hours of low irradiance light PER1 protein was nearly undetectable in the SCN of wildtype mice (Fig. 10B, left). This unexpected and previously un-reported finding contrasts sharply with the finding that in USP2^−/−^ mice PER1 protein was on the rise over this time course in either light or darkness (Fig. 10B, right). The decline in PER1 in low irradiance light in wildtype mice is not consistent with a direct effect of USP2 on PER1 since USP2 would be expected to have a stabilizing effect. It is generally thought that increasing PER1 is required for light-induced phase shifts. Our data suggest that at low light levels, which normally have little or no effect on phase in wild type animals, PER1 expression is actually declining. In contrast, the increased Per1 pre-mRNA (Fig. 10A) and rising PER1 protein (Fig. 10B) in USP2^−/−^ animals suggests that expression of PER1 is enhanced during the low light signal, which in this case leads to a phase delay. This suggests a scenario in USP2^−/−^ mice in which increased turnover of BMAL1 coincides with increased CLOCK/BMAL1-driven gene expression in the USP2^−/−^ SCN.

Although we often correlate protein abundance and stability with functional activity, our data on BMAL1 are consistent with other work indicating that it is the ubiquitinylated form of BMAL1 that is transcriptionally active [10] and that transcriptional activity is closely coupled to BMAL1 turnover (Fig. 10C). [9], [10]. Hence, we suggest that increasing expression of Per1 and other CLOCK/BMAL1 transcripts in USP2^−/−^ mice compared to wild type following low light might account for the enhanced phase delays observed in USP2^−/−^ mice.

## Discussion

Our principal finding is that mice lacking the de-ubiquitinylating enzyme USP2 are hypersensitive to light at the day-to-night transition, resulting in enhanced phase delays measured by wheel running activity. Notably, the sensitivity to early evening light exposure in USP2^−/−^ mice is specific to light beginning at ZT12 and occurs at scotopic irradiances that do not normally cause phase delays in wildtype animals. This suggests that USP2 negatively regulates its targets in the light-entrainment pathway, specifically in response to day lengthening at low irradiance levels corresponding to dusk. In other words, at least one USP2 target reduces the sensitivity to light.

In theory USP2 could regulate sensitivity to light by altering the response of the SCN to its light input or further upstream within the retina or retinohypothalamic tract. Our finding that USP2b is in complex with several clock components in the SCN, including PER1 and BMAL1 suggests that altered expression of clock components might account for the observed increased sensitivity to light in USP2^−/−^ mice. For example, altered clock function in the USP2^−/−^ mice might make them more susceptible to entrainment signals at the day to night transition. Of particular relevance is our finding that USP2b alters BMAL1 abundance and turnover. Consequently, decreased BMAL1 abundance and increased turnover in the absence of USP2 could account for the altered expression of clock controlled genes and core clock components observed. Furthermore, our finding that BMAL1/USP2 double heterozygotes exhibit enhanced phase shifts in low irradiance light suggests that the enhanced sensitivity phenotype seen in USP2^−/−^ mice could be mediated at least in part by BMAL1. We propose that BMAL1 mediates this effect through its interaction with CLOCK. It is of interest that a similar phase shift paradigm in CLOCK^−/−^ mice causes a phase advance rather than delay [20].

How could reduced abundance and increased turnover of BMAL1 enhance sensitivity to light? It has been reported that the transcriptional activity of BMAL1 (e.g., CLOCK/BMAL1) is closely coupled to its ubiquitinylation and turnover [10]. In this regard, BMAL1 is one of a long list of transcription factors in which transcriptional activity is closely coupled to ubiquitinylation [21], [22], [23], [24], [25]. In these cases transactivation is tightly controlled because the transcription factor itself is quickly degraded immediately following trans-activation. In a preliminary evaluation of this proposition we found that USP2^−/−^ mice exhibited 2–4 fold increases in the un-spliced, pre-mRNAs of three CLOCK/BMAL1 controlled genes (*Per1*, *Dbp*, and *Rev-erbα*) following low irradiance light and these changes were not seen in wildtype controls. We speculate that in the absence of USP2b, BMAL1 turns over more rapidly and that its ubiquitinylated form is transcriptionally active prior to degradation (Fig. 10C). This scenario becomes apparent in response to low irradiance light at ZT12, when USP2b expression is normally on the rise. The consequence is that targets of BMAL1/CLOCK transactivation are enhanced in the absence of USP2b. Among these are PER1 and PER2 as well ryanodine receptors [26], all of which are required for early evening phase delays.

Our data suggest that a functional relationship between USP2 and BMAL1 within the SCN could explain the enhanced phase delays observed in USP2^−/−^ mice. For example, early evening phase shifts are thought to depend on light-induced changes in intracellular Ca^++^ within SCN, which results from BMAL1- dependent transactivation, to control the abundance of ryanodine receptors in SCN neurons [26]. However, we cannot rule out the possibility that USP2 functions via regulation of BMAL1 at another site within the retinohypothalamic tract because both BMAL1 and USP2 are expressed in the retina and SCN. Furthermore, other targets of USP2 within retina or SCN could play a role in resetting. Currently, USP2a is known to de-ubiquitinylate fatty acid synthase [27], MDM2 and MDMX (mouse double minute 2 and X) [28] while USP2b is known to de-ubiquitinylate the epithelial sodium channel in kidney [29] and BMAL1 (our data). Other targets are possible within the clockwork because USP2b is present in protein complexes containing clock proteins. Although our initial survey suggests that neither CLOCK nor PER1 are USP2b targets, other clock components were not systematically evaluated. Our work has additionally focused on USP2b because of our initial finding of its effect on BMAL1 stability. USP2a, however, is also a clock-controlled protein that could control additional elements of the molecular clockwork.

The lowest irradiance level used in our experiments (∼0.05 µW⋅cm^−2^) is in the scotopic range and does not cause a phase delay in wildtype mice in the paradigm used in this study. This implies that light signaling normally associated with rod photoreceptors can cause phase resetting within the SCN and that USP2 is a component of a gating system that regulates sensitivity of the clockwork to early evening light. Recent studies in mice show that rods mediate entrainment in the scotopic range via their signaling through ipRGCs to the SCN [30], [31]. The implied gating system could operate at the level of the SCN, within the retinohypothalamic tract or both. At the level of the SCN de-ubiquitinylation of BMAL1 by USP2b in wild type animals would be expected to reduce BMAL1-dependent transactivation. Since this would include reductions in Per1 and in the activity of ryanodine receptors [26], which are required at the level of the SCN for light induced phase delays, we speculate that the gating system operates at least in part at the level of the SCN to render it less sensitive to its photic input. Alternatively, a USP2-mediated activity within the retina could regulate or filter signaling by rods through the system if ipRGCs. Either or both gating systems could be of adaptive significance in rodents at dusk by enabling a transition from cone-mediated day-time vision to rod-mediated night-time vision without undue alteration of clock phasing through use of a rod pathway to the SCN.

## Materials and Methods

### Animals

Construction of the *Usp2* targeting vector and the general strategy for introducing LoxP sites upstream and downstream of Exons 3–4 as well as upstream of the thymidine kinase (TK) neomycin (NEO) cassette (Fig. 2) was based on a previously published targeting vector strategy [39]. The resulting targeting construct was electroporated into R1 ES cells and after selection of G418 resistant ES cell clones with correct targeting, the clones were transiently transfected with a plasmid encoding Cre recombinase [39]. This resulted in clones carrying a conventional KO allele with a single LoxP site (Fig. 2A) and a “floxed” allele with LoxP sites in intron 2–3 and 4–5 with deletion of the TK-Neo cassette as verified by Southern blotting. These clones were injected into blastocysts in the Medical College of Wisconsin transgenic facility. Germ line transmission was obtained only for the conventional KO allele (Fig. 2A–C).

All mice were maintained according to a protocol approved by Institutional Animal Care and Use Committee at the Medical College of Wisconsin (Protocol Number AUA00000032). Male USP2^+/+^ or USP2^−/−^ mice were crossed with C57BL/6 mice and were housed in LD conditions (ZT0 = 0600 hrs; ZT12 = 1800 hrs). Male BMAL1^+/−^ mice from Jackson Laboratories were crossed with USP2^−/−^ females. Mice were euthanized at indicated times by CO_2_ (1L/min) followed by cervical dislocation.

### Behavioral analysis

Wheel running experiments involved males (1–11 months old); in each experiment the mice were age-matched. Mice were housed individually in running wheel cages (Actimetics Inc, Evanston, IL) on a 12-hour LD schedule with *ad libitum* feeding. Room fluorescent lighting produced light levels of 60–120 µW/cm^2^ depending on cage position, as measured with a radiometer (International Light, Newberryport, MA) from within the cage. In the constant light (LL) paradigm fluorescent light was attenuated to 5–20 µW/cm^2^ and every 10 days this was increased sequentially to 30–60, 50–100, 60–120, and 100–160 µW/cm^2^. In phase shift experiments each cage was equipped with a white LED that produced an un-attenuated irradiance (I) at the bottom, center of the cage of ∼9 µW⋅cm^−2^ (i.e., Log I 5.0) and neutral density filters (Kodak) were used to attenuate irradiance to ∼4.5 (Log I 4.5), ∼0.9 (Log I 4.0), ∼0.1 (Log I 3.0) and ∼0.05 µW⋅cm^−2^ (Log I 2.5).

Wheel running activity was recorded and analyzed using ClockLab™ software (Actimetrics, Evanston, Il). Phase shifts were calculated as the difference between the average onsets of activity 5 days following the experimental light treatment and the average onset of activity for the preceding 4 days. Mice were re-entrained to 12-hour LD for 7 days before each subsequent experimental light treatment.

### Tissue Explants

SCN regions were removed by placing the brain in a matrix device (Zivic Instruments, Pittsburgh, PA). 1 mm coronal sections of the hypothalamus were removed and 0.5 mm×0.5 mm explants representing the SCN were micro-dissected and either immediately frozen in liquid Nitrogen for protein analysis or in RNA Later (Qiagen) for RNA analysis. Five SCN regions were pooled for western blotting and one SCN region was used for each RT-qPCR run. Retinal explants were obtained by micro-dissection and immediately frozen in liquid nitrogen for western blotting (10 retinas/sample) or stored in RNA Later for RNA analysis (2 retinas/per sample). Liver was explanted (1 mm×1 mm), stored in RNA later, and used for RT-qPCR. RT-qPCR using Qiagen reagents and a Bio-Rad iCycler PCR unit was conducted according to detailed methods and with gene specific primers described below.

### Quantitative RT PCR

Ten mg of tissue in RNA later (Qiagen) was transferred to 500 µl of lysis buffer (RNeasy kit, Qiagen) and disrupted with a Pellet Pestle homogenizer (Kontes) for 1 minute followed trituration with an 18-gauge needle for 1 minute. The homogenate was transferred to a Qiashredder spin column (Qiagen) and spun at maximum speed for 2 minutes. RNA was isolated with an RNeasy kit (Qiagen) according to the product protocol. Total RNA was measured by spectrophotometry and diluted with ultra pure H_2_O to a final concentration of 0.05 µg/µl. Resultant cDNA using iScript cDNA synthesis kit (Bio-Rad) was diluted 1:5 in ultra pure H_2_O and 5 µl was used per 25 µl total RT-qPCR reaction (Bio-Rad iCycler). Each reaction was performed at 95° for 3 minutes, followed by 38 cycles of 30 seconds at 95°, annealing temperature for 30 seconds, and 20 seconds at 72°. The optimized annealing temperature for each primer pair was calculated from the mean melting temperature of the primer pair. Forward and reverse primers for *Usp2* were:

*Usp2a forward* 5′-GTCCCCGTCCCTGCTGCTCTCCAC-3′,

*Usp2a* reverse 5′- CATCTGTCGCCCCTTTTCTTCATCA-3′

*Usp2b forward 5′-* GGCCGCCCCCTGCTGAGAT-3′,

*Usp2b* reverse 5′- GAACGGCTGGCTGCTTGGTAGAGG-3′.

Primers were optimized and the slopes of the standard curves were ∼3.2 ± 0.5 with correlation coefficients equaling 0.99.

The remaining PCR primer sequences were obtained from previously published work: *Clock* [32], *Bmal1* [*33*], *Per1* [*34*], *Per2* [35], *Cry1, Rev-erbα, Dbp* [36], *pre-Per1* (courtesy of Charles Weitz, Harvard Medical School), *pre-Fos* [37], *pre-Dbp, pre- Rev-erbα* [38], and *RNA Polymerase II* [39]. RT(−) controls in each PCR sample used were negative. RT-qPCR products were quantified using the comparative ΔCT method described previously [40].

### Western blotting and Immunoprecipitations

Tissue samples were homogenized (1 min), triturated with an 18-gauge needle (1 min) and sonicated (2 sec) on ice in lysis buffer containing 75 mM Hepes (pH 7.4), 1.5 mM EGTA, 150 mM KCl, 1.5 mM MgCl_2_, 15% glycerol, 0.05% NP-40, and EDTA-free complete protease inhibitor cocktail (Roche). This was repeated twice and samples were then spun at 10,000 x g for 10 minutes at 4°C. The supernatant (lysate) was used for either western blotting or immunoprecipitation (IP). For IP 200–300 µg of protein was incubated on ice for 30 minutes with 50 µl of protein G beads or protein A beads conjugated to the appropriate antibody DMP (Sigma). After spinning beads and protein lysates at 10,000 x g (10 min) at 4°C, the lysate was incubated on ice with the appropriate IP antibody (30 min) while the beads were washed. The cleared lysates, beads and antibodies were incubated, rotating, overnight at 4°C, spun at 10,000 x g (30 sec) and washed 4 times with PBS. IPed complexes were eluded by boiling (10 min) in 50 µl of 6X sample buffer followed by a spin (5 min) at 10,000 x g. Twenty µg of total protein or 10–15 µl of immunoprecipitated protein sample was loaded onto pre-cast 10% denaturing gels (Bio-Rad) for all western blots. Transfers were with a small Genie electrophoretic device (Idea Scientific, Minneapolis, MN) and proteins were detected by ECL or by irradiance detection using an Odyssey imaging system (LI-COR Biosciences).

### Antibodies

We generated an antiserum against the C-terminal region of USP2 in guinea pig (Covance) that recognizes both USP2a and USP2b. Anti-Bmal1 antiserum (guinea pig) and PER1 (rabbit 1177) was kindly provided by Marina Antoch (Roswell Park Cancer Institute, Buffalo, NY) and David Weaver (UMass Medical Center, Worcester, MA) respectively. Other antibodies were obtained commercially as follows: anti-Bmal1 (rabbit, Abcam), anti-CK1ε (rabbit, Abcam), anti-Cry1 (mouse, Alpha Diagnostics International), anti-EGFR (rabbit, Abcam), anti-Per1 (rabbit, Pierce), anti-USP2 (rabbit, Abgent), γ-Tubulin (mouse, Abcam), anti-Ubiquitin (rabbit, Thermo Scientific), anti-HA (rabbit, Santa Cruz), anti-Flag (mouse, Sigma), and anti-c-MYC (mouse, Sigma).

### Cell Culture and Transfection

A cDNA encoding *mClock* was provided by Charles Weitz (Harvard Medical School), and *mBmal1* and *mPer2* cDNAs were provided by Marina *Antoch (Roswell Park Cancer Institute, Buffalo, NY). mPer1 and HA*-Ubiquitin cDNAs were purchased from Addgene. The cDNA for *mUsp2a* and *mUsp2b* were cloned from mouse (MGI:1858178). The Gateway cloning system (Invitrogen) was used to clone the cDNAs into the pCMV-BICEP-4 bicistronic vector (Sigma), which was converted to a Gateway destination vector. All constructs were verified by sequencing and western blotting following transient transfection in NIH3T3 or HEK293 cells obtained from the private ATCC Biology Collection.

NIH3T3 or HEK293 cells were cultured in DMEM (Sigma) supplemented with 10% Cosmic serum, 4 mM L-glutamine, 1 mM sodium pyruvate, and 1% pen/strep were transfected at approximately 80% confluency with Lipofectamine reagent (Invitrogen) and Plus reagent (Invitrogen). For protein stability assays HEK293 cells were transfected with 1 µg of either FLAG-GFP/MYC-CLOCK, -BMAL1, or -PER1 and increasing amounts (from 0 µg to 1 µg) of FLAG-USP2b/MYC-GFP. Protein analysis was done 24 hours following transfection. For protein turnover assays HEK293 cells at about 80% confluence were transfected with a bicistronic vector expressing either FLAG-USP2b/MYC- BMAL1, MYC-CLOCK, or MYC-PER1. Control vectors included the same MYC-proteins with FLAG-GFP. Cycloheximide (20 µg/ml) treatment began 24 hours following transfection and extracts were western blotted 0, 1, 2, 4, and 8 hours later.

### Statistical analyses

Usp2 rhythmicity at the level of the mRNA was tested using one-way ANOVA. Two-way ANOVA was used to test differences in phase shifts at various irradiances between wildtype and either USP2^−/−^, USP2^+/−^, BMAL1^+/−^, or USP2^+/−^/BMAL1^+/−^ mice and phase shifts measured in mice of different genotype at specific irradiance levels were compared to wildtype using an un-paired t-test. Period measurements and activity counts were compared between wildtype and USP2^−/−^ mice using an un-paired t-test. Two-way ANOVA was used to compare either mRNA or protein expression over time between wildtype and USP2^−/−^ mice. GraphPad Prism software (GraphPad Software, Inc) was used for both statistical analysis and plotting of quantitative data.
